# Method dependence, observer variability and kidney volumes in radiation dosimetry of ^177^Lu-DOTATATE therapy in patients with neuroendocrine tumours

**DOI:** 10.1186/s40658-015-0127-y

**Published:** 2015-10-24

**Authors:** Mattias Sandström, Ezgi Ilan, Anna Karlberg, Silvia Johansson, Nanette Freedman, Ulrike Garske-Román

**Affiliations:** Section of Nuclear Medicine and PET, Department of Surgical Sciences, Uppsala University, Uppsala, Sweden; Section of Medical Physics, Department of Immunology, Genetics and Pathology, Uppsala University, 751 85 Uppsala, Sweden; Section of Oncology, Department of Immunology, Genetics and Pathology, Uppsala University, Uppsala, Sweden; Medical Biophysics and Nuclear Medicine, Hadassah-Hebrew University Medical Center, Jerusalem, Israel

**Keywords:** Method dependence, Dosimetry, ^177^Lu-DOTATATE, Neuroendocrine tumours

## Abstract

**Background:**

Radionuclide therapy can be individualized by performing dosimetry. To determine absorbed organ doses in ^177^Lu-DOTATATE therapy, three methods based on activity concentrations are currently in use: the small volume of interest (sVOI) method, and two methods based on large VOIs either on anatomical CT (aVOI) or on thresholds on functional images (tVOI). The main aim of the present work was to validate the sVOI in comparison to the other two methods regarding agreement and time efficiency. Secondary aims were to investigate inter-observer variability for the sVOI and the change of functional organ volumes following therapy.

**Methods:**

Thirty patients diagnosed with neuroendocrine tumours undergoing therapy with ^177^Lu-DOTATATE were included. Each patient underwent three SPECT/CT scans at 1, 4 and 7 days after the treatment. Three independent observers calculated absorbed doses to the right and left kidney and the spleen using sVOI and one observer used aVOI. For tVOI, the absorbed doses were calculated based on automatically drawn isocontours around the organs at different thresholds (42, 50, 60 and 70 %). The inter-observer difference between the calculated absorbed doses for sVOI was calculated, and the differences between the three methods were computed. Ratios of organ volumes acquired at days 1, 4 and 7 versus the volume at day 1 were calculated for the tVOI method.

**Results:**

The differences in results of the absorbed dose calculations using all the sVOI and tVOI were small (<5 %). Absorbed dose calculations using aVOI differed slightly more from these results but were still below 10 %. The differences between the three dose calculation methods varied between <5 and 10 %. The organ volumes derived from the tVOI were independent of time for the spleen while they decreased with time for the kidneys. The fastest analysis was performed with the sVOI method.

**Conclusions:**

All three dose calculation methods rendered comparable results with small inter-observer differences for sVOI. Unlike the spleen, the functional volume of the kidneys decreased over time during therapy, which suggests that the absorbed dose calculation for the kidneys on activity concentrations should be performed for each time point. The sVOI is the preferred method for calculating absorbed doses in solid organs.

## Background

During the last decades, cancer survival in general has improved [1]. Several reports suggesting improved outcome in patients responding to PRRT have been published [2–4]. This is true for all treatment modalities including surgery, chemotherapy and radiation therapy. It has been shown that patients with somatostatin receptor positive neuroendocrine tumours can be treated with good results using peptide receptor radionuclide therapy (PRRT) with ^177^Lu-DOTA-D-Phe1-Tyr3-octreotate (^177^Lu-DOTATATE) [3–7]. The therapeutic effect is correlated to the delivered absorbed dose to the tumours for external beam radiation therapy (EBRT) and the same effect has been shown also for systemic treatment with ionizing radiation resulting from PRRT using ^90^Y-[DOTA]-D-Phe1-Tyr3-octreotide (^90^Y-DOTATOC) [8], and more recently in the case of pancreatic NETs using ^177^Lu-DOTATATE [9]. To maximize the tumour effect, the absorbed dose in the tumours should be as high as possible, while at the same time the absorbed doses to normal organs should be kept within the safety margins. The possibility of increased administered activity in therapy with ^177^Lu-DOTATATE is often limited by concern about toxicity resulting from radiation damage to healthy organs (mainly the bone marrow and kidneys) [4, 10–14]. An earlier study with 2 Gy as upper limit to the bone marrow and 23 Gy to the kidneys indicated that in most cases, the kidneys are the dose-limiting organs [15]. In two recent studies the first by Sabet et al. [16] on nephrotoxicity after PRRT with ^177^Lu-DOTATATE with accumulated administered activities from 14.8 to 37.8 GBq, only one of the 74 patients showed significant renal toxicity (≥grade 3). In the second study by Bodei et al. [17] in 290 patients receiving PRRT with ^177^Lu-DOTATATE with accumulated administered activities from 1.1 to 49.2 GBq, none of the patients showed significant (≥grade 3) nephrotoxicity while nine showed significant (≥grade 3) haematological toxicity. This indicates a potential to increase the administered activity further for individual patients.

Twenty-three Gy is the limit generally accepted for the absorbed dose to the kidney in external beam radiotherapy, based on the observation that TD5/5 (the absorbed dose where 5 % of the patients developed radiation nephritis) for the whole kidney is 23 Gy [18]. Due to inhomogeneous distribution at a microscopic level in PRRT with ^177^Lu-DOTATATE, higher absorbed doses can probably be accepted, and Konijnenberg et al. [19] argued for a limit of 29 Gy to the kidneys. In view of the low-dose rate and the ongoing continuous radiation in PRRT, the absorbed dose limits may be even higher. A biological effective dose (BED) up to 45 Gy has been suggested [20]; however, the influence of clinical risk factors on kidney tolerance has been reported [12, 14] and might be taken into account in the future.

In order to estimate the absorbed dose in the risk organs in PRRT with ^177^Lu-DOTATATE, the activity distribution and kinetics over time are needed. Since it is generally difficult to free-project the kidneys from surrounding organs and tumours in NET patients receiving therapy with ^177^Lu-DOTATATE, dosimetry using whole body imaging (2D) is insufficient [21]. Therefore, robust methods to calculate organ doses based on 3-D imaging need to be applied.

As previously demonstrated in a simulation study [22] and clinically [23], the absorbed dose for the kidneys originates mainly from the organs themselves (self-dose) because almost 90 % of ^177^Lu decay is by emission of short-range beta particles. Only a few percent of the absorbed dose result from the cross-dose from gamma radiation from the surrounding organs. The self-dose therefore yields a very good estimate of the absorbed dose and can be calculated by multiplying the activity concentration for an organ at the given time point by an almost size-independent activity concentration dose factor (ACDF), as published earlier [15, 21]. Since absorbed dose is absorbed energy per mass and the energy deposition from ^177^Lu generally can be assumed to be absorbed locally, it is of uttermost importance to know the concentration of decays, not only the total number of decays in an organ, at each time point. It has been demonstrated earlier [20, 21] that the estimated volumes of the kidneys in the patients receiving therapy with ^177^Lu-DOTATATE can have a large intra-patient variability depending on the method used to determine the volume.

Different methods can be used to measure organ activity concentrations from single photon emission tomography (SPECT) with low-dose computed tomography (CT) for attenuation correction (SPECT/CT). The most frequently used method is to delineate the whole organ either in the anatomical (CT) or the functional (SPECT) image. A second method, introduced in earlier reports [15, 21], is to measure the concentration using a small VOI with a specified volume yielding the activity concentration in a defined small volume within the organ. Comparison between dosimetry based on manually delineated whole organ VOIs versus small VOIs on SPECT images has shown acceptable agreement [21].

In times where financial resources are limited and time is a valuable resource, it is of importance that the calculation of the absorbed dose can be performed as fast as possible. For this reason, the setup time to calibrate the equipment has to be taken into account as well as the time spent for the dose calculation.

The main aim of this work was to compare dosimetry based on the small VOI (sVOI) method versus threshold VOI on functional images (tVOI) method and VOIs on anatomical CT images (aVOI) method. Secondary aims were to investigate the inter-observer variability in the sVOI method, the impact of the threshold applied in the tVOI method, the changes in functional volume (the volume where the ^177^Lu is located in the organ) over time from treatment, to study the differences in kidney volume estimated by tVOI and aVOI in patients receiving ^177^Lu-DOTATATE therapy, and to determine the time needed to perform the absorbed dose calculation using the different methods. All methods were applied to both kidneys and spleen in order to examine whether any differences between methods were related to the complex structure of the kidneys or method dependent.

## Methods

### Patients

Thirty patients (17 female and 13 male) with metastatic somatostatin receptor-expressing neuroendocrine tumours treated with ^177^Lu-DOTATATE were included in this study and all patients met previously described inclusion criteria [21]. Due to adjacent tumour uptake that made the tVOI method impossible to use, one left kidney and three right kidneys were excluded for all methods. For the anatomical CT images method, one more patient was excluded due to missing CT data.

^177^LuCl3 was purchased from IDB, and DOTATATE was a generous gift from Erasmus Medical Centre, Rotterdam.

### Compliance with ethical standards

There is no external funding, and all authors declare no conflict of interest.

Since September 2010, all patients have been included after giving their written informed consent into a prospective study (EudraCT no. 2009-012260-14) approved by the Regional Ethical Review Board in Uppsala. Before that time, patients were admitted after giving their informed consent on a single-patient basis for compassionate use with individual permission of the Swedish Medical Products Agency.

### Image acquisition

On all 30 patients, SPECT/CT of the abdomen was performed 1, 4 and 7 days after administration of the first therapeutic cycle of 7.4 GBq ^177^Lu-DOTATATE. Imaging was performed on an Infinia (International General Electric, General Electric Medical Systems, Haifa, Israel) dual-headed gamma camera equipped with 3/8” NaI(Tl)-crystals with VPC-5 (MEGP) collimators. A 20 % energy window was placed around the dominant 208.4 keV gamma ray energy of ^177^Lu to make the measurements. SPECT/CT of the upper abdomen including organs at risk (kidneys, liver and spleen), applying 120 frames with a 30-s exposure time per frame (total acquisition time for SPECT is then 30 min), was performed. For reconstruction, the Ordered Subsets Expectation Maximisation (OSEM) algorithm included in the Xeleris 3.0 workstation (International General Electric, General Electric Medical Systems, Haifa, Israel) was used with earlier default settings (iterative reconstruction with eight subsets and four iterations followed by a Hann filtering with a cut-off of 0.85). The images were attenuation corrected with the concomitantly CT-created attenuation map acquired on a four-channel CT scanner (Hawkeye, 140 kVp, 3.0 mA and half rotation).

### Image analysis

All images were analysed by the 3 methods sVOI, aVOI and tVOI.

#### sVOI method

Analysis was performed by three observers using small spherical volumes of interests (VOIs; 4 ml) in both kidneys and spleen, using in-house developed software within the Hermes platform on a Hermes HNAC workstation with Gold 2.9 (HERMES, Stockholm, Sweden). Observers were instructed to place VOIs over the region of highest activity in the healthy organ, as described previously [21].

#### tVOI method

Threshold measurements were performed for both kidneys and spleen using 42, 50, 60 and 70 percent of the maximum in the organ as limit. The 42 % threshold has previously showed to most closely resemble the true volume [24], and the higher thresholds were chosen to see what impact a higher threshold would have on the quantification. The threshold measurements were performed using the VOI tool in NEDPAS Software Tools [25] Version Built 26042009 written by R. Boellaard, VU University Medical Center, Amsterdam, The Netherlands.

#### aVOI method

The organs were delineated on the CT images using Dosimetry toolkit software included in the Xeleris 3.0 workstation (International General Electric, General Electric Medical Systems, Haifa, Israel). We attempted to use a threshold on the Hounsfield scale for the delineation on the CT images. However, the delineation according to the Hounsfield scale did not yield acceptable anatomic delineation, so the final organ definition was completely manual.

Calibration of SPECT images was based on a 100-ml sphere containing a known activity concentration. The sphere was placed inside a thorax phantom, which was scanned repeatedly (18). The sensitivity of the camera is independent of measurement technique, still the sensitivity factor to be used will be different for all techniques and cut-off levels since the proportion of the peripheral parts of the volume (where edge effects take place) that are included is method/cut-off dependent.

Phantom measurements filled with a high activity concentration imaged weekly for 10 weeks confirmed that there were no dead time issues in the patient measurements.

Activity concentrations were determined for all of these observers/methods for each time point, and time-integrated activity concentration was calculated as the area under the curve of a single exponential fit (from injection start to infinity) to the time–activity concentration curve. In the 42, 50, 60 and 70 % threshold VOIs, the measured functional volumes were determined separately for each time point while for the anatomical (CT) images the volumes were defined on the 24-h images and transferred to the 3-day and 7-day images, so only one volume was determined and used for purposes of calculating organ activity concentration. Changes between functional volumes obtained at different thresholds and different time points were calculated as ratios relative to the 1-day image.

### Absorbed dose calculations

Absorbed doses to both kidneys and spleen were calculated by multiplication of time-integrated organ activity concentration with the appropriate ACDF, thus considering only the self-dose.

### Time to perform an absorbed dose calculation

The time required to calculate the absorbed dose for each of the three methods was measured from start of calculation until the report was completed.

### Statistical methods

A Wilcoxon matched-pair signed rank test was used to test for a statistically significant difference between the organ volumes at different time points estimated by the tVOI method. *P* values less than 0.05 were treated as statistically significant and less than 0.0001 as highly statistically significant.

Absorbed dose data were analysed in two ways: 1) the percent difference between one observer/method versus another and 2) as a correlation coefficient between one observer/method versus another.

## Results

**Fig. 1:**
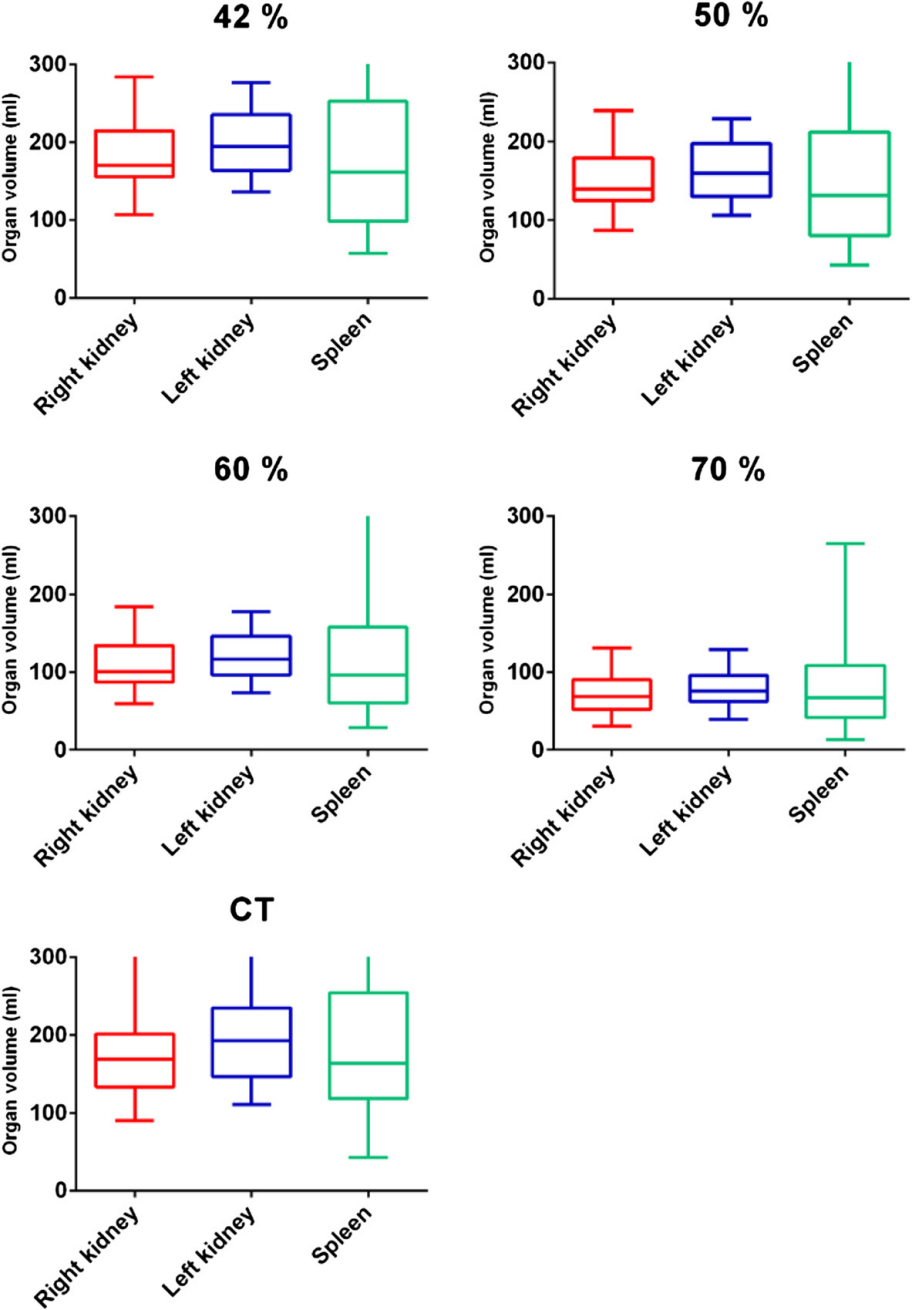
Organ volumes (ml) 1 day after treatment in the right kidney (*red*), left kidney (*blue*) and spleen (*green*) determined with functional images using 42, 50, 60 and 70 % cut-off and anatomical CT

Kidney and spleen volumes evaluated by tVOI and aVOI methods are presented as a box-whiskers plot in Fig. 1. The kidney volumes evaluated by using the threshold method with 42 % cut-off had a median (min–max) value of 170 (107–283) ml for the right kidney (26 patients) and 195 (136–276) ml for the left kidney (29 patients). With a threshold of 50 %, the calculated volume was 140 (87–239) ml for the right kidney and 160 (106–229) ml for the left kidney. The CT delineated volume was 169 (90–342) ml for the right kidney (26 patients) and 193 (111–315) ml for the left kidney (28 patients). There was no good correlation in the volumes of the left and right kidneys on an intra-patient basis. When significant differences in size and/or absorbed dose occurred, the maximum absorbed dose was used for limitation of the therapy.

**Fig. 2:**
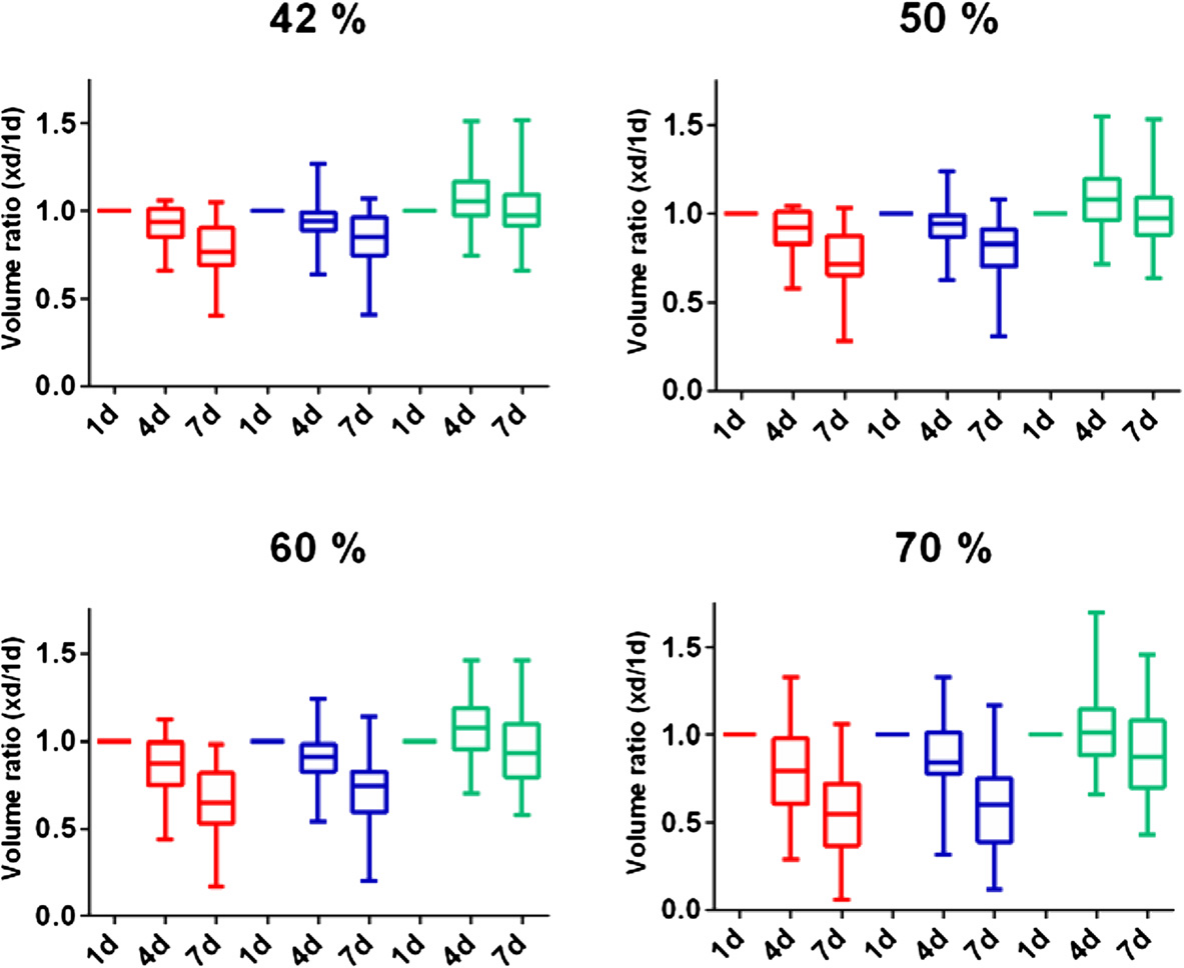
Ratio of volumes 1, 4 and 7 days versus the day-1 volume in the right kidney (*red*), left kidney (*blue*) and spleen (*green*) using 42, 50, 60 and 70 % cut-off

Figure 2 presents a box-whiskers plot of the changes in the functional volume and Table 1 shows the percent difference in the functional volume of kidneys and spleen over time using the threshold method with the different cut-offs (42, 50, 60 and 70 %). For the spleen, no obvious difference was seen between the 1-, 4- and 7-day measurements besides a small increase from day 1 to day 4 that resolved at day 7. For the kidneys, a consistent decrease of the functional volume over time was observed, independent of the cut-off level. There was a large patient variability and a clear difference depending on the applied cut-off. *P* values showing significant and highly significant differences are indicated in Table 1.

**Table 1:** Percentage difference between volumes for different cut-offs using the tVOI method. Data are presented as median (min; max)

	Days	42 %	50 %	60 %	70 %
Right kidney	1 vs 4	−6.6 (−34.1; 6.1)*	−7.8 (−42.8; 4.3)**	−12.3 (−55.6; 12.2)**	−20.7 (−70.8; 32.9)**
	1 vs 7	−23.3 (−60.5; 5.0)**	−27.8 (−71.8; 3.3)**	−35.2 (−83.5; −1.5)**	−44.7 (−93.6; 5.9)**
	4 vs 7	−15.5 (−40.1; 15.3)**	−17.3 (−51.1; 16.3)**	−24.7 (−63.7; 13.0)**	−29.3 (−80.9; 38.3)**
Left kidney	1 vs 4	−5.9 (−35.8; 26.8)**	−6.0 (−37.0; 23.8)**	−8.9 (−46.3; 24.0)**	−15.9 (−68.2; 33.2)*
	1 vs 7	−14.7 (−59.0; 7.1)**	−17.0 (−69.4; 7.6)**	−25.3 (−79.9; 13.7)**	−39.5 (−88.4; 17.3)**
	4 vs 7	−9.0 (−44.5; 26.5)**	−11.8 (−55.1; 19.0)**	−17.0 (−65.9; 20.6)**	−27.4 (−76.3; 37.0)**
Spleen	1 vs 4	5.5 (−25.5; 50.9)*	8.1 (−27.6; 55.5)*	7.5 (−30.5; 46.0)*	1.3 (−33.6; 70.1)
	1 vs 7	−2.1 (−33.8; 52.0)	−2.4 (−36.4; 53.3)	−6.1 (−41.9; 46.2)	−12.4 (−56.8; 46.2)*
	4 vs 7	−6.6 (−33.7; 24.6)*	−9.6 (−36.7; 21.8)**	−13.4 (−48.7; 27.9)**	−16.0 (−57.3; 48.7)**

Two-tailed *P* values from the Wilcoxon matched-pair signed rank tests between the functional volume measurements was also calculated to evaluate if the differences were significant

*P values lower than 0.05 were significant; **P value lower than 0.001 was highly significant

The differences in organ volume presented above result in differences in organ activity concentrations and consequently have implications for absorbed dose estimates.

**Fig. 3:**
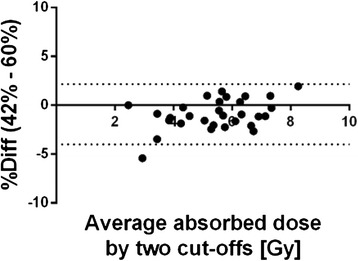
Percentage difference against mean for two cut-off values using tVOI method calculating absorbed dose to the right kidney

In Fig. 3, an example of the difference in the absorbed dose calculations between two cut-offs (42 versus 60 %) using the tVOI method is presented as a Bland-Altman plot. Percentage differences in the absorbed dose calculations for all cut-off values used in the tVOI method are presented in Table 3. Figure 3 shows the tendency to slightly lower absorbed dose when using 42 % rather than 60 % threshold, confirmed in Table 3. However, all correlation coefficients were 0.99 and above. Despite the small differences, there was a good agreement between the different cut-off levels (less than 5 %).

**Fig. 4:**
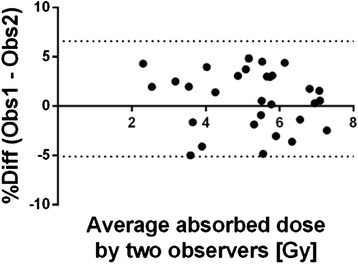
Percentage difference against mean for two observers calculating absorbed dose to the right kidney using sVOI method

Percentage differences in the absorbed dose calculations for all three observers using the sVOI method are presented in Table 2 and for observer 1 versus observer 2 as a Bland-Altman plot in Fig. 4. The dashed lines in the Bland-Altman plots correspond to 1.96 SD corresponding to double-sided *P* = 0.05. All correlation coefficients were 0.96 and above. Inter-observer variability in percentage difference is generally low (less than 5 %) showing a good agreement between the different observers using the sVOI method for absorbed dose calculations.

**Table 2:** Percentage difference between absorbed dose calculations for different observers using the sVOI method. Data are presented as median (min; max)

		Observer 2	Observer 3
Right kidney	Observer 1	0.8 (−4.8; 5.0)	3.6 (−4.5; 16.8)
	Observer 2		2.9 (−5.8; 18.7)
Left kidney	Observer 1	1.2 (−4.4; 9.6)	1.8 (−6.6; 12.7)
	Observer 2		0.7 (−8.9; 15.5)
Spleen	Observer 1	−0.6 (−7.4; 11.1)	0.0 (−16.0; 11.9)
	Observer 2		0.7 (−16.9; 9.6)

**Fig. 5:**
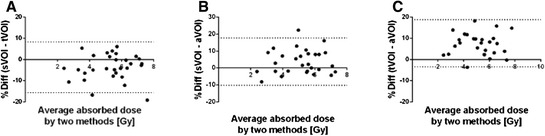
Percentage difference against mean in calculation of absorbed dose to the right kidney using different measuring techniques **a** sVOI vs. tVOI, **b** sVOI vs. aVOI and **c** tVOI vs. aVOI

Bland-Altman plots showing differences between all three methods (sVOI, tVOI and aVOI) for the right kidney are presented in Fig. 5. The percentage differences for all the organs are presented in Table 4. All correlation coefficients were 0.91 and above showing a good correlation. There is good agreement between the sVOI and tVOI methods (differences less than 10 %). In general, the aVOI method generated 5 to 10 % lower absorbed doses with differences up to 25 %.

The time to perform absorbed dose calculation measurements using different methods depended heavily on both patient and observer. The results can only be taken as an indication of the time needed to complete the calculation. The time required for both the sVOI and the aVOI to calculate the absorbed dose, including the left and right kidneys and spleen, ranged between 15 and 45 min, while for the tVOI this ranged between 3 and 5 h for the three organs independent of number of thresholds.

## Discussion

To our knowledge, the present work is the first study comparing inter-operator variability and method dependence for radiation dosimetry of ^177^Lu-DOTATATE in a group of patients in a systematic way including method-specific sensitivity (accounting for edge effects) in the calculations. We also believe this to be the first study investigating the changes in functional organ volume over time from administration of PRRT, with implications for dosimetry calculation. This study is not aiming at an analysis of absorbed doses to functional sub-units but calculation of average absorbed dose to the kidneys.

A range of publications during recent years has reported on the clinical value of radionuclide therapy directed towards somatostatin receptors for therapy of patients with neuroendocrine tumours [3, 4, 6, 13]. Still, many issues remain to be solved in order to improve and personalize the therapy [26]. In the literature, a general agreement on the importance of individualized therapy is noted, where dosimetry is one tool to achieve an individual risk-assessment for sensitive organs [27]. Kidney toxicity is a limiting factor for radionuclide therapy with somatostatin analogs, especially when using ^90^Y [4, 14, 20]. The observed kidney toxicity for therapy with ^177^Lu-DOTATATE is lower, with a maximum tolerable absorbed dose yet to be defined [3, 27].

The work of our own group has been dedicated to developing a robust and clinically applicable dosimetry protocol for solid organs based on 3-D imaging [15, 21]. In accordance with several other groups, we found ^177^Lu-DOTATATE PRRT dosimetry based only on 2-D scanning problematic due to inherent difficulties of delineating healthy organ uptake from tumours and physiological uptake as in the bowel [12, 28, 29].

Organ dosimetry in radionuclide therapy is highly dependent on the correct input data defined by activity concentrations measured over time. The volume calculation for kidneys has been problematic, and the results are highly dependent on the method applied [30]. The weight of normal kidneys varies with body surface and gender and is in the range of 130 to 160 g for men and 120 to 150 g for women [31]. The volume estimations from the aVOI and tVOI with 42 % and 50 % cut-off agree well with earlier published data on kidney volumes (range 100 to 300 ml) in patients with neuroendocrine tumours receiving PRRT [20, 21], and also with those achieved in healthy adults by using an MRI-based method, that showed high agreement with ex vivo data [30]. The estimated volumes by tVOI using 60 and 70 % cut-offs were too small to reflect the true volumes. By using dedicated contrast enhanced CT, the outlining of the kidneys should theoretically be more anatomically correct. However, in this study, the kidney volumes were calculated from low-dose CT performed for attenuation correction. Because the software used for the organ delineation is dependent on a distinct attenuation contrast between the kidney and surrounding adipose tissue that sometimes was hard to define, the resulting estimated CT volumes may therefore be less precise. Another limitation in this study was that the aVOI method was only performed once by one observer. The large volume differences between methods highlight the importance to find a method that can render activity concentrations without the necessity of defining the whole organ volume.

The changes over time in functional volume observed in the threshold measurements for the spleen were minor and not significant when comparing between day 1 and 7. For the kidneys, the ratios between late and early measurements after therapy showed a clear decrease for all cut-off levels. Our data suggest that the functional volume of the kidneys decreases over time during ^177^Lu-DOTATATE therapy, which is of clinical interest. In reports by other authors [28, 32, 33], the kidney volume has been defined at one time point and used as a correction for 2-D-based dosimetry calculations, assuming that the (functional) volume of the kidneys is constant during the whole radionuclide therapy. In the light of our reported findings, this assumption might for the majority of the patients lead to an underestimation of the absorbed dose to the kidneys. The sequential decrease in functional volume influences the calculation of the absorbed dose considerably and might result in an absorbed dose difference of about 10 % and sometimes even more. In patient dosimetry, this fact introduces another factor of uncertainty in the dose calculation of the kidneys, besides the above-mentioned inherent problems when only 2-D data are used.

The reason for the observed volume change during therapy can only be speculated about. Increased blood flow to the kidneys as an immediate reaction to the therapy might be one contributing explanation, as can be the co-infusion of amino acids during therapy.

It is of note, though, that the different cut-offs (42, 50, 60 and 70 %) applied with tVOI resulted in almost the same absorbed dose. This indicates, that a precise estimate of the actual kidney volume is not necessary as long as the applied cut-off value is in the range of 42 to 70 %, the activity concentration is calculated for every time point and that the same cut-off is used for calibration.

Table 2 shows that the percentage difference between the absorbed dose calculations of the three different observers using the sVOI method is small, indicating a robust method. The differences are for most patients between 2 and 5 %, rarely above 5 % and only occasionally up to 10 % in patients with organs close to adjacent tumours that make the placing of the small volumes problematic/uncertain. Our results show that the use of sVOI method in this setting renders an operator-independent result. Generally, there were small, 5 to 10 %, differences in the absorbed dose calculations by using the tVOI method and the various cut-offs (Table 3). In this study, large VOIs give slightly higher absorbed doses than the small VOI method. This might seem strange since small VOI placement was directed to include the highest possible value within the organ without including tumour tissue. One possible explanation might be that parts of the small 4 cm^3^ VOI was influenced by partial volume effect (PVE) in patients with thinner parenchyma rims, which was not a problem in the calibration setting. We found a good agreement between the calculated absorbed doses using the sVOI and the tVOI while the aVOI method rendered 5 to 10 % lower absorbed doses (Table 4 and Fig. 5). This agrees well with the observation of decreasing functional volume according to the tVOI method. The correlation coefficients range from 0.92 to 1.00 showing a strong correlation between the sVOI and the tVOI methods. The correlation coefficients between the above and data derived from aVOI method were slightly lower ranging from 0.86 to 0.98, but still showing a good correlation.

**Table 3:** Percentage difference in the absorbed dose calculations for different cut-offs using the tVOI method. Data are presented as median (min; max)

		50 %	60 %	70 %
Right kidney	42 %	0.9 (−0.6; 2.7)	−0.8 (−3.4; 1.9)	−2.8 (−5.8; 2.2)
	50 %		−1.7 (−2.9; −0.5)	−3.7 (−6.5; −0.5)
	60 %			−2.1 (−3.9; 0.3)
Left kidney	42 %	1.0 (−0.1; 2.0)	−0.4 (−2.4; 2.0)	−2.2 (−6.5; 1.5)
	50 %		−1.4 (−2.7; 0.0)	−3.2 (−6.8; −0.5)
	60 %			−1.9 (−4.2; −0.5)
Spleen	42 %	−1.0 (−0.7; 2.4)	−0.2 (−3.1; 3.0)	−1.4 (−5.4; 2.0)
	50 %		−1.2 (−3.1; 0.5)	−2.4 (−4.8; 0.0)
	60 %			−1.3 (−2.7; −0.1)

**Table 4:** Percentage difference between absorbed dose calculations methods (sVOI, tVOI and aVOI). sVOI represents sVOI method by observer 1, tVOI represents the 42 % level of the tVOI method and aVOI represents the CT-based volumes in the aVOI method. Data are presented as median (min; max)

		tVOI	aVOI
Right kidney	sVOI	−3.2 (−17.6; 6.3)	4.5 (−5.0; 25.0)
	tVOI		8.4 (−3.8; 22.9)
Left kidney	sVOI	−3.4 (−10.5; 5.5)	3.9 (−9.8; 24.8)
	tVOI		8.0 (−5.8; 29.5)
Spleen	sVOI	−3.2 (−21.3; 5.5)	−2.2 (−19.6; 21.9)
	tVOI		1.1 (−17.1; 19.9)

Dosimetry based on tVOI and sVOI takes into account possible volume changes during therapy and therefore seems preferable. The tVOI was by far the most time consuming (3–5 h per patient) while the other methods were much faster (15–45 min). For this reason, the sVOI method, in our hands, is the preferred method to calculate absorbed doses in solid organs.

## Conclusions

Volume-based absorbed dose calculations using sVOI method agree well with the tVOI method while the aVOI method results in slightly lower absorbed doses. In this study, the inter-observer variability using the sVOI was small and the functional kidney volume decreased between day 1 and 7 after therapy infusion with ^177^Lu-DOTATATE. We conclude that sVOI is the preferred method for calculating absorbed doses, since it both provides a robust tool for measuring volume concentrations over time in solid organs and is time efficient.
